# Factors associated with low birth weight at Debre Markos Referral Hospital, Northwest Ethiopia: a hospital based cross-sectional study

**DOI:** 10.1186/s13104-019-4143-1

**Published:** 2019-02-27

**Authors:** Animut Alebel, Fasil Wagnew, Cheru Tesema, Alemu Gebrie, Daniel Bekele Ketema, Getnet Asmare, Getiye Dejenu Kibret

**Affiliations:** 1 Department of Nursing, College of Health Science, Debre Markos University, P.O. Box 269, Debre Markos, Ethiopia; 2 Department of Public Health, College of Health Science, Debre Markos University, Debre Markos, Ethiopia; 3 Department of Biomedical Science, School of Medicine, Debre Markos University, Debre Markos, Ethiopia; 4 College of Health Science, Debre Tabor University, Debre Tabor, Ethiopia

**Keywords:** Low birth weight, Associated factors Debre Markos, Northwest Ethiopia

## Abstract

**Objective:**

To assess the prevalence and associated factors of low birth weight among newborns delivered at Debre Markos Referral Hospital, Northwest Ethiopia.

**Results:**

From the total of 368 newborn baby/mother pairs planned to be participated, 338 agreed and involved in the study giving a response rate of 91.2%. In this study, the prevalence of low birth weight was 21.6 (95% CI 17.5, 26%). Being rural residence (AOR 2.0, 95% CI 1.0, 4.1), duration of pregnancy (AOR = 7.6, 95% CI 3.3, 17.4), and having complications during pregnancy (AOR 2.6, 95% CI 1.2, 5.7) were found to be factors significantly associated with low birth weight.

**Electronic supplementary material:**

The online version of this article (10.1186/s13104-019-4143-1) contains supplementary material, which is available to authorized users.

## Introduction

Globally, low birth weight (LBW) continues to be a major public health problem and it is associated with a range of short-and long-term consequences. Overall, it is estimated that 15%–20% of all births worldwide are LBW, representing more than 20 million births a year [1]. The majority (96.5%) of LBW births were reported from low-and middle-income countries and especially, in the most vulnerable populations [2, 3]. The regional estimated prevalence indicated that 28% of LBW were found in south Asia and 13% were in sub-Saharan Africa [4]. As indicated by the 2016 Ethiopian Demographic and Health Survey (EDHS) report, 13% of newborn babies were low birth weight [5].

Low birth weight newborns are 20 times at higher risk of death as compared to normal birth weight newborns, unless interventions have been made [6, 7]. Besides, LBW newborns experienced a number of long and short-term complications. Some short term complications include recurrent apnea, intra and periventricular hemorrhage, lung hemorrhage, jaundice, infections, retinopathy of prematurity, perinatal asphyxia, hypothermia, hypoglycemia, anemia, and infection [8]. Long term consequences include hypertension, diabetic nephropathy, proteinuria, progressive renal disease at late age, deafness, neurologic complications like cerebral palsy, developmental delay with IQ less than 70, epilepsy and behavioral disturbance [9]. Regarding factors associated with LBW, the previous Ethiopian studies pointed out that maternal anemia, not attending antenatal care, having a history of abortion, being female newborn, not taking additional food during pregnancy, rural resident, lack of formal education, and complications during pregnancy were some of the factors significantly associated with LBW [10–20].

As an intervention, the Ethiopian government recognized LBW as a serious public health problem and implemented numerous strategies. In addition, nongovernmental organizations (CU-ICAP and WHO) and professional associations including the Ethiopian Pediatric Society have been working to tackle this problem [21, 22]. Current and up-to-date information regarding LBW are essential for policy makers to take appropriate actions. Therefore, this study aimed to identify factors associated with LBW at Debre Markos Referral Hospital. The findings of this study will highlight the prevalence and associated factors of low birth weight with implications to improve health workers’ interventions, to ensure cost-effectiveness, and to accelerate the reduction of neonatal mortality.

## Main text

### Study design, period and area

A hospital-based cross-sectional study was conducted from September 22 to October 25, 2017 at Debre Markos Referral Hospital, Northwest Ethiopia. Debre Markos town is located 300 km far away from Addis Ababa, the capital city of Ethiopia, and 256 km far from Bahir-Dar, the main city of Amhara Regional State. The hospital is expected to provide services for more than 3.5 million people in its catchment area. Apart from other services, the hospital provides ANC and delivery services for pregnant women.

### Population

All mothers who gave birth at Debre Markos Referral Hospital from September 22 to October 25, 2017 were our source population. All newborn babies consecutively delivered at Debre Markos Referral Hospital during the study period were included. However, newborn babies whose mothers suffered from severe medical (diabetes mellitus) or surgical condition, twin delivery, mothers with unknown last menstrual period (LMP) with the absence of ultrasound evidence at the time of the study period were excluded from the study.

#### Sample size determination and sampling technique

The required sample size was calculated by using a single population proportion formula, and calculated using Open Epi Version 3. To calculate our sample size, the following statistical assumptions were considered: P = prevalence of low birth weight 32.1% taken from a study conducted in Ethiopia [23], Z α/2 = the corresponding Z score of 95% CI and d = Margin of error (5%).

$${\text{N}} = \frac{{\left( {{\text{Z}}_{{\frac{\alpha }{2}}} } \right)^{2} \times {\text{P}}\left( {1 - {\text{P}}} \right)}}{{\left( {\text{d}} \right)^{2} }}$$

N = (1.96)^2^ *0.32*0.68/(0.05)^2^ ^=^ 334. Finally, after adding 10% of none response rate the final sample size of our study was calculated as 368. All 368 newborn babies consecutively delivered at Debre Markos Referral Hospital during the study period were planned to be included.

### Operational definitions

Low birth weight was defined as the neonate birth weight less than 2500 grams.

Birth interval was defined as the length of time between two successive live births.

### Data collection tool and procedure

Data were collected using an interviewer administered structured questionnaires. The questionnaire was prepared after reviewing similar studies conducted in Ethiopia, and Ethiopian demographic and health survey, 2016. It was first prepared in English language, then two language experts translated to Amharic, and translated back to English to check for consistency. To ensure data quality, a 1-day training was given regarding data collection tool and data collection process for both data collectors and supervisor. In the data collection process, three female intern nursing students were involved. Every day, at the end of the data collection period, all questionnaires were reviewed and checked for any errors by the supervisor and data collectors.

#### Data processing and analysis

All filled questionnaires were checked for completeness, consistency, accuracy and entered into Epi-Data Version 3.1 and then, exported to SPSS software version 22 for data analysis. Frequency, proportion, and summary statistics (mean and median) were used to describe the study population in relation to the relevant variables. Bivariable logistic regression analysis was used to check the association between dependent and independent variables. Then, in the bivariable analysis those variables having *p* value less than 0.2 were fitted into the multivariable logistic regression analysis for controlling the effect of confounders. Odds ratio with their 95% of CI was computed and variables having p-values less than 0.05 in the multiple logistic regression models were considered as significantly associated with the dependent variable.

### Results

#### Sociodemographic characteristics

From the total of 368 newborn baby/mother pairs planned to be participated, 338 agreed and involved in the study giving a response rate of 91.2%. The mean age of mothers was 29.9 years (SD = ± 6.7) and more than half of the respondents (61. 8%) were found within the age group of 21–34 years. Regarding religion, the majority (86.1%) of women are orthodox religion followers. Similarly, the majority (97.9%) of mothers were from Amhara Region. Concerning marital status, majority (93.5%) of the mothers were married, and about half (50.3%) of the mothers were from urban areas (Table 1).

**Table 1:** Socio-demographic characteristics of the mothers at Debre Markos Referral Hospital, Northwest Ethiopia, 2018

Variables (N = 338)	Category	Frequency (N)	Percent (%)
Age	< 20 years	29	8.6
	21–34 years	209	61.8
	≥ 35 years	100	29.6
Religion	Orthodox	291	86.1
	Others	47	13.9
Ethnicity	Amhara	331	97.9
	Others	7	2.1
Maternal educational status	Unable to read and write	141	41.7
	Able to read and write	197	58.8
Husband educational status	Unable to read and write	131	38.8
	Able to read and write	207	61.2
Residence	Rural	168	49.7
	Urban	170	50.3
Marital status	Married	316	93.5
	Others	22	6.5
Mothers occupation	Farmer	138	40.8
	Merchant	47	13.9
	Housewife	68	20.1
	Governmental employee	68	20.1
	Others	17	5

#### Obstetric related characteristics of the respondents

More than half of the respondents were multiparous. About two-third (71.3%) of the pregnancies were intended. With regard to current pregnancy related problems, only 77 (22.8%) developed the problems. Three hundred sixteen (93.5%) have no history of low birth weight. Two hundred eighty-nine (85.5%)) of the mothers had ANC follow up. Of which, 29 (8.6%) had four and above ANC follow up care (Table 2).

**Table 2:** Obstetric related characteristics of the respondents at Debre Markos referral hospital, Northwest Ethiopia, 2018

Variable (N = 338)	Category	Frequency (N)	Percent (%)
Parity	≥ 4	257	76
	< 4	81	24
Birth interval (in months)	< 24 months	50	14.8
	≥ 24 months	218	64.8
	Not applicable	70	20.4
Wanted pregnancy	Yes	241	71.3
	No	97	28.7
Pregnancy related problems	Yes	77	22.8
	No	261	77.2
Types of pregnancy problems	HTN	40	11.8
	PROM	18	5.4
	APH	5	1.5
	Anemia	14	4.1
Previous history of low birth weight	Yes	22	6.5
	No	316	93.5
Complications during the current Pregnancy	Yes	62	18.4
	No	276	81.6
Types of complication	HTN	53	15.7
	HIV	6	1.8
	Others	3	0.9
Malaria infection	Yes	7	2.1
	No	331	97.9
STIs for the current pregnancy	Yes	9	2.6
	No	329	97.9
Types of the problem	Vaginal discharge	6	1.7
	Spills	3	0.8
Anemia during pregnancy	Yes	50	14.8
	No	288	85.2
Duration of pregnancy	Preterm (< 37 weeks)	54	16
	Term (≥ 37 weeks)	284	84
ANC follow-up	Yes	289	85.5
	No	49	14.5
Number of ANC follow-up (N = 289)	1–4	266	92.0
	≥ 4	23	8.0
IFA supplementation	Yes	268	79.3
	No	70	21.7
Number of IFA tablet (N = 268)	< 60	174	66.9
	60–90	94	33.1

#### Nutritional, behavioral, and neonatal factors

Two hundred twenty-even (67.2%) of the mothers were counseled about dietary intake during antenatal care follow up. Ninety-two (27.2%) of them took extra meal during pregnancy. Three hundred thirty-six (99.4%) of the respondents did not smoke during pregnancy. Regarding alcohol drinking one hundred ninety-two (56.5%) of the respondents did not drink alcohol during their pregnancy. Among three hundred thirty-eight newborns 149 (44%) were females (Additional file 1: Table S1).

#### Prevalence of low birth weight

This study noted that the prevalence of low birth weight among newborn babies delivered at Debre Markos Referral Hospital was found to be 21.6% (95% CI 17.5, 26%). The mean birth weight of the neonates was 2945.6 g (SD ±  594.3 g).

### Bivariable and multivariable logistic regression analysis

In the multivariable logistic regression analysis, place of residence (being rural), having complications during the current pregnancy, and duration of pregnancy (being preterm) were found to be an independent predictors of low birth weight. Accordingly, residence of the mother was strongly associated with low birth weight; mothers living in the rural areas were 2.0 times more likely to have LBW babies when compared to those mothers who live in urban area (AOR 2.0, 95% CI 1.0, 4.1). In addition, mothers who had any form of complication during the current pregnancy were 2.6 times more likely to give LBW babies as compared to their counterparts (AOR 2.6, 95% CI 1.2, 5.7). Moreover, duration of pregnancy was also another strong predictor of low birth weight. From this finding, preterm (< 37 weeks) babies were 7.6 to have LBW as compared to term (≥ 37 weeks) (AOR 7.6, 95% CI = 3.3, 17.4) (Additional file 2: Table S2).

### Discussion

The overall prevalence of low birth weight noted in this study was found to be 21.6% (95% CI 17.5, 26%). This finding is in line with studies conducted in Gondar University Referral Hospital (17.1%) [10], Jimma Zone (22.5%) [12], and Southwest Ethiopia (17.88%) [11]. However, our finding is much higher than studies done in Selected Public Hospitals of Addis Ababa (8.8%) [14], Tigray, Northern Ethiopia (10.5%) [15], Adwa General Hospital (10%) [17], Axum and Laelay Maichew Districts (9.9%) [13], and 3 Zonal Hospitals of Tigray (14.6%) [16]. The possible explanation for these variations could be due to the differences in study settings. This study was conducted at a referral hospital where many of the pregnant women were referred from other districts and general hospitals because of high-risk pregnancy. Therefore, this factor could have a contribution for the high prevalence of LBW in the study area. The other possible explanation for the above variation might be the difference in sociodemographic characteristics of study participants. Moreover, this difference could be explained by the difference in time gap between these studies and seasons of the year as birth weight may have seasonal variations [24].

The study found that mothers from rural areas were more likely to deliver LBW baby as compared to their urban counterparts. This finding is consistent with studies conducted in Bale Zone, Southwest Ethiopia, and Tigray, Northern Ethiopia [15, 18]. However, our finding contradicts with a previous study conducted in Jimma Zone [12]. The difference might be due to inadequate intake of food during pregnancy among mothers in rural areas. Evidence suggested that maternal undernutrition is positively associated with LBW [25]. In addition, this could be due to the fact that medical services, health information, and nutritional awareness are more accessible to mothers who are residing in urban areas than rural counterparts. Moreover, rural mothers did not commonly visiting health institution, which results a greater risk of poor perinatal outcomes than urban mothers.

In this study, mothers who had complications during the current pregnancy were more likely to have LBW newborns as compared to mothers who hadn’t any pregnancy related complications. This finding contradicts with a previous study conducted in Ethiopia [15]. This study documented that maternal complication was significantly associated with increasing macrosomia. However, in our study maternal complications were significantly associated with increasing low birth weight. The possible explanation for these contradicted findings could be due to the difference in inclusion and exclusion criteria of our study participants. In our study, mothers who had history diabetes mellitus were excluded from the study. We excluded mothers who had diabetes mellitus from the study was due to the fact that diabetes mellitus is a known risk factor for macrosomia. Therefore, we believe that incorporating these mothers will give inappropriate results in the prevalence of low birth weight.

Furthermore, gestational age was also another important factor, which strongly associated with the occurrence of LBW. Likewise, newborn babies born before gestational the age of 37 weeks were more likely to be LBW as compared to that of newborn babies born at the gestational age of 37 weeks and above. This finding is in concordance with previous studies conducted in Ethiopia [12, 16, 20]. It is known that as the gestational age of the fetus falls below the acceptable range of time, body weight of the fetus falls dramatically due to prematurity. This study found that the prevalence of LBW at Debre Markos Referral hospital was higher as compared to previous studies. Duration of pregnancy, complication during the current pregnancy, and rural residence were found to be factors significantly associated with low birth weight.

## Limitations of the study

The study is a cross sectional study; therefore, it doesn’t show a cause and effect relationship as well as it doesn’t show seasonal variation in low birth weight. In addition, important factors like intrauterine infections during pregnancy, issues related to placental abnormalities and pre-pregnancy weight were not addressed due to lacking of data. Moreover, the study unable to include mothers who delivered at home because the study was conducted in the hospital.

## Additional files

**Additional file 1: Table S1.** Nutritional, behavioral and neonatal factors of the respondents at Debre Markos Referral Hospital Northwest Ethiopia, 2018.

**Additional file 2: Table S2.** Bivariable and multivariable logistic regression analysis of factors associated with low birth weight at Debre Markos Referral Hospital, Northwest Ethiopia, 2018.

## Abbreviations

ANC: antenatal care
EDHS: Ethiopian Demographic and Health Survey
IFA: iron and folic acid
LMP: last menstrual period
LBW: low birth weight
WHO: World Health Organization
