# Supplementary material for: Factors associated with low birth weight at Debre Markos Referral Hospital, Northwest Ethiopia: a hospital based cross-sectional study
Source: BMC Res Notes. 2019 Feb 27;12:105. doi: 10.1186/s13104-019-4143-1 (PMC6391783; doi:10.1186/s13104-019-4143-1)
Supplement: Supplementary file 1 — Additional file 1: Table S1. Nutritional, behavioral and neonatal factors of the respondents at Debre Markos Referral Hospital Northwest Ethiopia, 2018. [file 13104_2019_4143_MOESM1_ESM.docx]

**Table S1:** Nutritional, behavioral and neonatal factors of the respondents at Debre Markos Referral Hospital Northwest Ethiopia, 2018

| **Variable (N=338)** | **Category** | **Frequency (N)** | **Percent (%)** |
| --- | --- | --- | --- |
| **Nutritional counseling** | Yes | 227 | 67.2 |
|  | No | 111 | 32.8 |
| **Additional food** | Yes | 92 | 27.2 |
|  | No | 246 | 72.8 |
| **Cigarette smoking** | Yes | 2 | 6 |
|  | No | 336 | 99.4 |
| **Alcohol drinking** | Yes | 147 | 43.5 |
|  | No | 192 | 56.5 |
| **Birth weight in gm** | <2500 g | 73 | 21.6 |
|  | $\geq$2500 g | 265 | 78.4 |
| **Sex** | Female | 149 | 44.1 |
|  | Male | 189 | 55.9 |
| **Congenital problems** | Yes | 0 | 0 |
|  | No | 338 | 100 |
