# Supplementary material for: Factors associated with low birth weight at Debre Markos Referral Hospital, Northwest Ethiopia: a hospital based cross-sectional study
Source: BMC Res Notes. 2019 Feb 27;12:105. doi: 10.1186/s13104-019-4143-1 (PMC6391783; doi:10.1186/s13104-019-4143-1)
Supplement: Supplementary file 2 — Additional file 2: Table S2. Bivariable and multivariable logistic regression analysis of factors associated with low birth weight at Debre Markos Referral Hospital, Northwest Ethiopia, 2018. [file 13104_2019_4143_MOESM2_ESM.docx]

**Table S2.** Bivariable and multivariable logistic regression analysis of factors associated with low birth weight at Debre Markos Referral Hospital, Northwest Ethiopia, 2018

| **Variables** | **LBW** | | **COR (95% CI)** | **AOR (95%CI)** |
| --- | --- | --- | --- | --- |
|  | **Yes (N)** | **No (N)** |  |  |
| **Residence** |  |  |  |  |
| Rural | 49 | 119 | 2.5 (1.5, 4.5) | **2.0 (1.0, 4.1) **** |
| Urban | 24 | 146 | 1 | **1** |
| **Marital status** |  |  |  |  |
| Marriage | 65 | 251 | 1 | **1** |
| Others | 8 | 14 | 2.2 (0.9, 5.5) | 1.7 (0.6, 5.4) |
| **Complications during pregnancy** |  |  |  |  |
| Yes | 23 | 39 | 2.6 (1.5, 4.8) | **2.6 (1.2, 5.7) **** |
| No | 50 | 224 | 1 | **1** |
| **Duration of pregnancy** |  |  |  |  |
| Preterm (<37 weeks) | 25 | 29 | 6.3 (3.4, 11.8) | **7.6 (3.3, 17.4) **** |
| Term ($\geq$ 37 weeks) | 44 | 239 | 1 |  |
| **Iron in number** |  |  |  |  |
| <60 | 40 | 134 | 1.9(0.9, 3.7) | 1.5 (0.7, 3.3) |
| 60-90 | 13 | 81 | 1 |  |
| **Nutritional counseling** |  |  |  |  |
| Yes | 39 | 34 | 1 |  |
| No | 188 | 77 | 0.5(0.3, 0.8) | 0.6 (0.3, 1.5) |

** Factors significantly associated with low birth weight
